# Leaving no one behind: Towards equitable global elimination of hepatitis C

**DOI:** 10.7189/jogh.10.010308

**Published:** 2020-06

**Authors:** Elin Hoffmann Dahl, Hassaan Zahid, Khawar Aslam, Wasim Jafri

**Affiliations:** 1 Norwegian Centre for Elimination of Hepatitis C, Lovisenberg Diakonale Hospital, Oslo, Norway; 2 Médecins Sans Frontières (MSF), Oslo, Norway; 3 Medical Doctor, Karachi, Pakistan; 4 Aga Khan University, Karachi, Pakistan

Until the 1950s, tuberculosis (TB) was a public health threat in many high-income countries, however, since the development of a vaccine, introduction of effective treatments and rising living standards, incidence has plummeted and new cases are now predominantly imported cases from endemic regions [1]. In predominantly poorer countries, TB still represents a major challenge. The stark global health inequity is highlighted by the 2015 World Health Organisation (WHO) ‘*End TB’* strategy which seeks to *“eliminate TB as a public health problem”* by 2050 [2] – a century after the disease came under control in high income countries. Today, the growing incidence of multidrug resistant (MDR-TB) and totally drug resistant (XDR-TB) are adding to the complexity of disease control; limited funds, that could once have gone directly into public health programs, must now be redirected to research and development for new drugs to tackle drug-resistant disease. This provides a cautionary tale for hepatitis C elimination.

At the discovery of the “non-A, non-B hepatitis” virus in 1989, later named hepatitis C (HCV), this disease was unknown and untreatable, and many naturally considered the diagnosis a death sentence. Interferon provided some respite, but it came at a huge cost for patients who experienced the toxic combination of a prolonged course of injected therapy with extensive side-effects, and ineffective viral clearance with frequent relapse. Treatment was revolutionized in 2011 with the introduction of oral, direct-acting antivirals (DAAs) which have vastly shorter treatment courses and virtually no side-effects. Viral clearance increased from around 50% with injected interferon to over 90% on an oral DAAs. This development has been a pivotal in the control of hepatitis C.

In many countries with well-functioning health services, HCV is already limited to subgroups of the population. Countries such as the United Kingdom, Georgia and Egypt are well on their way to reaching the WHO goals of elimination by 2030 [3]. Most other countries are lagging behind, particularly those low and middle-income countries (LMIC) with a high prevalence. HCV is still a menace in these countries because of poor health care systems, the need for universal screening and the challenge of competing priorities with inadequate resources. In Pakistan, the country with the second largest burden of hepatitis C, prevalence data from recent years has shown around a 40% increase in HCV antibody rates in the general population. Not only are the health systems ill-equipped to provide testing and treatment services to those suffering from HCV, high rates of nosocomial transmission from poorly functioning health care facilities add to the complexity of the outbreak [4].

Whilst more than 80 countries have developed national plans to eliminate HCV, fewer than half have followed up good intentions with the necessary financial commitment to eliminate the disease [5]. The global resurgence in HCV control has been driven by the shared promise of new treatments, and outrage at exorbitant drug prices, which rich and poor nations alike struggled to afford. Hepatitis C has, for a brief period in time, been “everyone’s problem”. In a few years HCV could be another disease of the poor, restricted to low and middle-income countries alone. So how can we stop HCV from going down the same path as TB, requiring a renewed global elimination strategy 50 years down the line?

**Figure:**
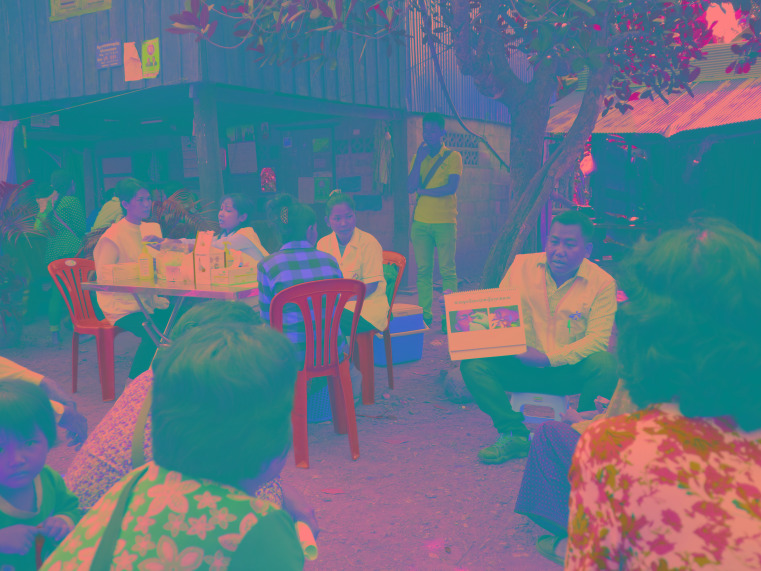
Photo: Simon Ming/MSF (used with permission).

##  “WHAT’S YOUR STATUS?”

While price was once the limiting factor blocking access to DAAs, with increasing availability of affordable generics this is no longer the case in many countries. Though falling prices have increased treatment uptake, the major challenge in tackling the HCV epidemics is not only access to treatment or affordability, but a lack of knowledge: 90% of people living with viral hepatitis are unaware of their status.

In high prevalence settings where the HCV antibody prevalence exceeds WHO thresholds (2% or 5% in different contexts), screening of the general population is a dire need. In practice, this means that during all interactions with the health care system clients should be asked: “Do you know your HCV status?”. By integrating HCV screening into all existing health programs this avoids missed opportunities for screening, increases cost-efficiency and takes advantage of other operational structures already in place, including TB and HIV programmes. The bottom line is that we need to diagnose and treat at least 5 million people worldwide every year in order to reach the WHO elimination goals by 2030 [6].

## SIMPLIFIED DIAGNOSTIC AND TREATMENT ALGORITHMS – TIME TO SCALE UP

The diagnosis and follow up of patients with HCV has been simplified by pan-genotypic drug regimens which no longer require genotyping and laboratory follow up testing, and the validation of low-cost approaches to the assessment of liver status (ie, APRI score). Furthermore, clinical trials have shown that treatment can be delivered effectively by trained nurses in community settings [7] and shortened treatment durations, down to 8 weeks for most patients, mean more patients can be treated within the same service capacity.

Currently, confirmation of viral clearance following treatment is recommended for all patients. With the increasing efficiency of new combinations of DAAs – approaching 100% – we question whether an additional polymerase-chain reaction (PCR) test is cost-effective from a public health perspective. The WHO elimination goal of 90% reduction in prevalence can be achieved with a low failure rate. Cost-benefit analyses could help establish the optimal use of resources of particular importance in low-resource settings. Pooled procurement of diagnostics may also bring down the price of PCR cartridges, as has been done for TB.

We believe that as with other infectious diseases focus must be put on contact-tracing with the implementation of “a household model”, whereby screening and treatment of the whole household as a unit is routinely undertaken when one household member is positive. Household members are likely to share many of the same risk factors thought to spread disease, eg, local practitioners, barbers and hygiene products. This is an established concept within infectious disease approaches to preventing re-infections and missed opportunities within communities of people who inject drugs.

## THE TIME IS NOW

Despite huge progress, many unanswered questions remain in the DAA era. Insufficient focus has been given to the potential rise in resistance to DAAs; many continue to consider the threat of drug resistance to be negligible despite growing evidence to the contrary [8]. Particularly in low-resource settings, the lack of generic options for treatment failure cases means that there is a clear theoretical potential for drug-resistant HCV strains to spread in the community, creating problems in years to come. Few predicted the rapid rise of MDR-TB when multiple drug-regimens were made available 60 years ago, nor the evolution of XDR-TB. If hepatitis C becomes a disease of the poor as was once the case of TB, history reminds us there will be little financial incentive for research and development of new drugs and progress can be lost.

Prevention must remain at the heart of tackling the HCV epidemic. Lifting people out of poverty and eliminating the spread of disease though health services, including local practitioners, is therefore key to successfully tackling HCV. We advocate for the integration of educational material into educational curricula in high prevalence countries. Patient Ambassadors may help in reducing the stigma of this now easily curable disease.

The scale of the HCV epidemic demands national ownership of elimination programs, including strong financial commitment. Treatment of all patients with chronic HCV has been shown to not only be cost-effective, but cost-saving in the long run as an earlier intervention prevents the development of cirrhosis [9]. Others can learn from Egypt, the country with the world's largest HCV disease burden. The problem has been a high priority for national government and the country is making remarkable progress in reaching the WHO elimination goals through driving down drug prices, optimizing procurement processes, improving infection control and blood safety, introducing mass education programs, improving community engagement, and implementing widespread testing with linkage to free treatment and follow-up care [10].

National governments and global health communities must recognize the risk of history repeating itself and not allow hepatitis C to follow the same path as TB. With incidence of HCV on the rise, the role of drug resistance still unknown and the importance of poverty alleviation underplayed, the warning signs are there to see. Our time is now. Let us combine the fantastic opportunity presented by the development of direct-acting antivirals with knowledge sharing, national leadership and the necessary financial commitments, to meet the WHO goal of eliminating hepatitis C as a public health threat globally by 2030.
